# Effects of venlafaxine on the expression level and methylation status of genes involved in oxidative stress in rats exposed to a chronic mild stress

**DOI:** 10.1111/jcmm.15231

**Published:** 2020-04-13

**Authors:** Paulina Wigner, Ewelina Synowiec, Piotr Czarny, Michal Bijak, Paweł Jóźwiak, Janusz Szemraj, Piotr Gruca, Mariusz Papp, Tomasz Śliwiński

**Affiliations:** ^1^ Laboratory of Medical Genetics Faculty of Biology and Environmental Protection University of Lodz Lodz Poland; ^2^ Department of Medical Biochemistry Medical University of Lodz Lodz Poland; ^3^ Department of General Biochemistry Faculty of Biology and Environmental Protection University of Lodz Lodz Poland; ^4^ Department of Cytobiochemistry Faculty of Biology and Environmental Protection University of Lodz Lodz Poland; ^5^ Institute of Pharmacology Polish Academy of Sciences Krakow Poland

**Keywords:** brain structures and blood cells, chronic mild stress model, gene expression and methylation, oxidative and nitrosative stress, rat, venlafaxine

## Abstract

Recent human and animal studies indicate that oxidative and nitrosative stress may play a role in the aetiology and pathogenesis of depression. This study investigates the effect of chronic administration of the serotonin‐norepinephrine reuptake inhibitor, venlafaxine, on the expression and methylation status of *SOD1*, *SOD2*, *GPx1*, *GPx4*, *CAT*, *NOS1* and *NOS2* in the brain and blood of rats exposed to a chronic mild stress (CMS) model of depression. Separate groups of animals were exposed to CMS for 2 or 7 weeks; the second group received saline or venlafaxine (10 mg/kg/d, IP) for 5 weeks. After completion of both stress conditions and drug administration, the mRNA and protein expression of selected genes and the methylation status of their promoters were measured in peripheral mononuclear blood cells (PBMCs) and in brain structures (hippocampus, amygdala, hypothalamus, midbrain, cortex, basal ganglia) with the use of TaqMan Gene Expression Assay, Western blot and methylation‐sensitive high‐resolution melting techniques. CMS caused a decrease in sucrose consumption, and this effect was normalized by fluoxetine. In PBMCs, *SOD1*, *SOD2* and *NOS2* mRNA expression changed only after venlafaxine administration. In brain, *CAT, Gpx1, Gpx4* and *NOS1* gene expression changed following CMS or venlafaxine exposure, most prominently in the hippocampus, midbrain and basal ganglia. CMS increased the methylation of the Gpx1 promoter in PBMCs, the second *Gpx4* promoter in midbrain and basal ganglia, and *SOD1* and *SOD2* in hippocampus. The CMS animals treated with venlafaxine displayed a significantly higher CAT level in midbrain and cerebral cortex. CMS caused an elevation of Gpx4 in the hippocampus, which was lowered in cerebral cortex by venlafaxine. The results indicate that CMS and venlafaxine administration affect the methylation of promoters of genes involved in oxidative and nitrosative stress. They also indicate that peripheral and central tissue differ in their response to stress or antidepressant treatments. It is possible that that apart from DNA methylation, a crucial role of expression level of genes may be played by other forms of epigenetic regulation, such as histone modification or microRNA interference.

These findings provide strong evidence for thesis that analysis of the level of mRNA and protein expression as well as the status of promoter methylation can help in understanding the pathomechanisms of mental diseases, including depression, and the mechanisms of action of drugs effective in their therapy.

## 1 INTRODUCTION

Depression is the most common of all mental disorders, with around 350 million sufferers Worldwide. According to WHO estimation, depression will become the second most common health problem in the World by 2020.^1^, ^2^ Furthermore, patients with depression are characterized by an increased risk of the development of somatic disease, including cardiovascular disease,^3^ obesity,^4^ diabetes,^5^ cancer^6^ and cognitive impairment.^7^ Despite intensive research, the ethology and pathogenesis of depression remain unclear.^8^ Moreover, around 30% of depressed patients do not benefit from antidepressant drug treatment.^9^, ^10^ Unfortunately, despite extensive studies, the pathophysiology of depression is not fully known, because the condition is not associated with any gross alternations of brain.^11^

On the other hand, a rapidly growing body of evidence suggests the involvement of various factors in the pathophysiology of depression, including inflammation, DNA damage and impairment of tryptophan transformation. There are also reports suggesting that affective disorders, including depression, are characterized by disturbances of the oxidant/antioxidant balance, that in consequence lead to damage of cellular macromolecules.^12^, ^13^, ^14^, ^15^, ^16^, ^17^ Reactive oxygen and nitrogen species can cause changes in cell membranes, receptor and enzymes functions, as well as activity of genes, and these imbalances can be influenced by antidepressant treatments.^14^, ^18^, ^19^ Among the signs of antioxidant defects, increased levels of 8‐F2‐isoprostane (8‐iso‐PGF2α), malondialdehyde (MDA) and 8‐hydroxy‐2‐deoxyguanosine have been found in depressed patients and are also believed to be markers of the effectiveness of an antidepressant treatment.^20^, ^21^, ^22^, ^23^ For example, an enhanced concentration of MDA is normalized after 3 months of SSRI treatment.^24^, ^25^ Similarly, chronic mild stress (CMS) model was associated with increased protein peroxidation—carbonyl—level in prefrontal, hippocampus, striatum and cortex as compared to control rats.^26^ Moreover, MDA level was increased in cerebellum and striatum of stressed rats.^26^ However, in another study, bupropion or sertraline increased 8‐iso‐PGF2α in patients with major depression and the severity of the disease was strongly correlated with an increased level of F2 isoprostane.^27^

Decreased levels of enzymatic and non‐enzymatic antioxidants can also play a crucial role in the mechanisms of depression. For example, a low glutathione peroxidase (Gpx) and superoxide dismutase (SOD) activity have been observed in depressed patients, with this decrease being correlated with the severity of the disease^28^, ^29^, ^30^, ^31^; however, opposite effects have also been reported.^25^, ^32^, ^33^ Additionally, antidepressant therapy may lead to partly improve of antioxidant response.^33^ Similarly, previous studies found that chronic ozone inhalation induced depression‐like symptoms, including anxiety, and reduced cortical and hippocampal SOD and CAT activity.^34^, ^35^ Interestingly, next study showed that the antidepressant therapy caused a reduction myeloperoxidase activity in the amygdala, hippocampus, amygdala and prefrontal cortex while SOD and CAT activity is believed to increase in nucleus accumbens of rat brain after antidepressant therapy.^36^

Another important oxidative enzyme associated with depression is xanthine oxidase (XO); a post‐mortem study had shown an increased level of XO in serum and the thalamus.^37^, ^38^ In addition, patients with depression have been characterized by decreased levels of vitamin E, vitamin C, zinc, uric acid, coenzyme Q10 and glutathione (GSH), although the latter did not change following electroconvulsive therapy.^19^, ^32^, ^39^, ^40^, ^41^, ^42^, ^43^, ^44^ Moreover, an animal study showed that the level of GSH was reduced in cerebral cortex, hypothalamus and brain stem regions of stressed mice, although it was not changed in cerebellum.^45^

Nitric oxide (NO) is another important chemical associated with the mechanisms of depression. Low levels of NO help facilitate the release of dopamine and noradrenalin, but high concentrations lead to nitration and hypernitrosylation of amino acids and proteins. This results in the generation of highly reactive substances: NO‐tyrosine, NO‐tryptophan and NO‐arginine.^46^, ^47^ It was found that the levels of IgM against NO‐aspartate, NO‐phenylalanine and NO‐tyrosine are increased in serum of depressed patients and the inhibition of NO synthesis can induce an antidepressant‐like effect.^48^ Thus, a NO inhibitor can increase the effectiveness of serotonergic antidepressants (SNRIs) and can be applied to patients with treatment‐resistant depression.^48^ Furthermore, an animal study suggests that the depression may be associated with increased activity of endothelial NOS (eNOS) and increased nNOS (neuronal NOS; NOS1) protein and mRNA expression in the hippocampus.^49^ However, Yoshino et al^50^ found that antidepressant treatment increased *nNOS* mRNA expression in hippocampus, midbrain, cerebellum and olfactory bulb, and iNOS (inducible NOS, NOS2) mRNA expression in frontal cortex and midbrain, and decreased *eNOS* mRNA expression in most brain regions.

The above data suggest that the mechanisms of depression can be associated with disturbances in the balance between oxidants and antioxidants. Thus, antioxidant agents may be an effective antidepressant therapy. Molecular hydrogen has antioxidative activities, and the mice after inhalation of hydrogen were characterized by decreased pathological damage, neuronal apoptosis and BBB disruption and reversed the cognitive decline.^51^ Similarly, Gao et al^52^ found that that repeated inhalation of hydrogen‐oxygen mixed gas decreased both the acute and chronic stress‐induced depressive‐ and anxiety‐like behaviours of mice. The next antioxidant compound—vanillin—inhibits the protein oxidation and lipid peroxidation in hepatic mitochondria. Thus, many previous studies showed that the vanillin relieved symptoms of CMS and it may be a potential antidepressant.^53^, ^54^, ^55^ Moreover, Amira et al^55^ found that CMS procedure caused an increase of lipid peroxidation and a decrease of GSH and serotonin in the brain. Sesamol is another antioxidant agent, which exerted antidepressant‐like effects, since it reversed the unpredictable chronic stress‐induced behavioural, including increased immobility period and reduced sucrose preference and biochemical parameters (increased lipid peroxidation and nitrite levels; decreased GSH levels, SOD and catalase activities) in stressed mice.^41^ Human studies also confirmed that antioxidants, including N‐acetylcysteine, may relieve symptoms of depression.^56^ On the other hand, a growing body of evidence suggests that antidepressants, including SSRIs, serotonin norepinephrine reuptake inhibitors (SNRIs) and tricyclic antidepressants (TCAs), may have antioxidant action.^57^ Therefore, a chronic treatment of imipramine increased SOD and CAT activity and decreased lipid and protein damage in prefrontal cortex and hippocampus of rats.^58^ Similarly, Zafir et al^59^ found that the activities of SOD, CAT, GST, GR and GSH levels in the rat brain increased after fluoxetine and venlafaxine administration. Additionally, the therapy prevented lipid and protein oxidative damage induced by stress.

Therefore, this study aimed to investigate whether: (a) the CMS procedure, used as an validated animal model of depression^59^, ^60^, ^61^ changes the expression of *SOD1*, *SOD2*, *GPx1*, *GPx4*, *CAT*, *NOS1* and* NOS2* at the mRNA and protein levels in peripheral blood mononuclear cells (PBMCs) and is selected brain structures (hippocampus, amygdala, midbrain, hypothalamus, cerebral cortex and basal ganglia); (b) chronic administration of serotonin‐norepinephrine reuptake inhibitor, venlafaxine, affects the expression of these genes; (c) the CMS procedure and venlafaxine administration cause epigenetic changes, that is methylation level of these gene promoters; (d) a degree to which these changes in methylation affect the genes expression; and (e) the changes observed in PBMCs can serve as markers of similar changes in the brain. The last point has an important clinical implication, as there is a great need for peripheral markers that would allow earlier diagnosis, more precise prognosis of pharmacotherapy outcome, and more personalized therapies of the mood disorders. All of the genes analysed in our study are located on chromosomes significantly associated with depression (Table S1).

## 2 MATERIALS AND METHODS

### 2.1 Animals

The study was carried out on male Wistar Han rats (Charles River), brought into the laboratory for adaptation to housing conditions 1 month before the start of the experiment. Except as described below, the animals were singly housed with freely available food and water, and maintained on a 12‐hour light/dark cycle (lights on at 8.00) in a constant temperature (22 ± 20°C) and humidity (50 ± 5%) conditions. All procedures used in this study conform to the rules and principles of Directive 86/609/EEC and have been approved by the Bioethical Committee at the Institute of Pharmacology, Polish Academy of Sciences, Krakow, Poland.

#### 2.1.1 Chronic mild stress procedure

The CMS procedure was conducted as previously described.^60^, ^61^, ^62^ Briefly, the animals (200‐220 g) were first trained to consume a 1% sucrose solution in seven once weekly baseline tests, in which sucrose solution was presented for 1 hour following 14‐hour food and water deprivation in the home cage. Subsequently, sucrose consumption was monitored once weekly, under similar conditions, until the end of the study. On the basis of their sucrose intakes in the final baseline test, the animals were divided into two matched groups. One group was exposed to the stress procedure for a period of 2 or 7 weeks. Each week of the stress regime consisted of two periods of food or water deprivation, two periods of 45‐degree cage tilt, two periods of intermittent illumination (light on and off every 2 hours), two periods of soiled cage (250 mL water in sawdust bedding), one period of paired housing, two periods of low intensity stroboscopic illumination (150 flashes/min), and three periods of no stress. The duration of all stressors was 10‐14 hours, and they were used individually and continuously, day and night. The control, non‐stressed animals were housed in separate rooms, with no contact with the stressed animals. They were deprived of food and water for 14 hours before each weakly sucrose test, but otherwise food and water were available ad libitum. In all CMS studies conducted in our laboratory, the stressed animals display a gradual decrease in the consumption of the sucrose solution to approximately 40% of pre‐stress values. When this effect stabilized, that is after initial 2 weeks of stress, the animals were either decapitated (see below) or further divided into matched subgroups, and for subsequent 5 weeks, they received daily administration of vehicle (1 mL/kg, IP) or venlafaxine (10 mg/kg, IP). The drug was administered to all animals (ie control and stressed) at approximately 10.00, and the weekly sucrose tests were carried out 24 hours after the last dose.

Twenty‐four hours after the sucrose test conducted following initial 2 weeks of stress, that is before venlafaxine administration was commenced (groups: Control and Stressed) and after the final sucrose test conducted following 7 weeks of stress, that is after completion of 5 weeks administration (groups: Control/Venla, Stressed/Saline, Stressed/Venla), the animals were decapitated and the blood and brain samples were collected (see below).

### 2.2 Specimen collection and separation of peripheral blood mononuclear cells

Blood samples were collected into 5 mL vacutainers containing EDTA. Peripheral blood mononuclear cells (PBMCs) were isolated based on differential migration of cells during centrifugation, that is mixed with equal volumes of PBS, layered on top of Gradisol L (Aqua‐Med) and centrifuged. The interfacial layer (lymphocyte coat) was transferred to a new tube and centrifuged. The supernatant was removed and PBMCs stored as pellets at −20℃ until required.

### 2.3 Isolation of RNA and DNA from PBMCs

RNA and DNA were extracted from PBMCs using the commercial spin column methods and eluted in RNAse‐Free water (GenElute Mammalian Total RNA Miniprep Kit; Sigma‐Aldrich; QIAamp DNA Mini Kit; Qiagen, respectively), in accordance with the manufacturer's protocols. Total concentrations of RNA and DNA were determined by spectrophotometer. The purity of the RNA and DNA samples was determined as a 260/280 nm OD ratio with expected values between 1.8 and 2.0. Finally, the RNA and DNA samples were stored at −20°C until required for further analysis.

### 2.4 Specimen collection and RNA/DNA isolation from animal brain

After blood harvesting, hippocampus, amygdala, midbrain, hypothalamus, cerebral cortex and basal ganglia were removed, rapidly frozen in liquid nitrogen and stored at −80°C until further analysis. A sufficient volume of PBS was added to each samples, which were then homogenized by a FastGene^®^Tissue Grinder (Nippon Genetics Europe). The homogenized samples were sonicated, centrifuged and rinsed with PBS using a commercial kit (ISOLATE II RNA/DNA/Protein Kit; Bioline), according to the manufacturer's instructions. Finally, the total concentration and purity of the RNA and DNA isolated from each sample was determined using a spectrophotometer as a 260/280 nm OD ratio with expected values between 1.8 and 2.0. Finally, the RNA and DNA samples were stored at −20°C until further analysis.

### 2.5 Reverse transcription and gene expression

The reverse transcription reaction was performed using a High‐capacity cDNA Reverse Transcription Kit (Applied Biosystems). Briefly, the following components were mixed to form a 20 µL reaction volume: nuclease‐free water; 10xRT Buffer; 10xRT Random Primers; 25xdNTP Mix (100 mM); total RNA (0.5 ng/µL) and MultiScribe^®^ Reverse Transcriptase. The reaction tubes were incubated for 10 minutes at 25°C, then for 120 minutes at 37°C, and then finally for 5 minutes at 85°C to inactivate reverse transcriptase. PCR was performed in a C1000™ programmed Thermal Cycler (Bio‐Rad Laboratories Inc). After the reverse transcription, the cDNA samples were stored at −20°C until further analysis.

Expression of SOD1 (assay ID: Rn00566938_m1), SOD2 (assay ID: Rn00690588_g1), Gpx1 (assay ID: Rn00577994_g1), Gpx4 (assay ID: Rn00820818_g1), CAT (assay ID: Rn00560930_m1), NOS1 (assay ID: Rn00583793_m1) and NOS2 (assay ID: Rn00561646_m1) genes was performed on a TaqMan Gene Expression Assay in a CFX96™ Real‐Time PCR Detection System Thermal Cycler (Bio‐Rad Laboratories Inc). The house‐keeping gene for human 18S ribosomal RNA gene (18S) was used as an internal control (reference gene) for all reverse transcription‐quantitative polymerase chain reactions (RT‐qPCRs), as it normalizes RNA input measurement errors and variations in reverse transcriptase‐PCR efficiency.

For the PCR process, a 10 µL mixture was used consisting of total cDNA samples, a TaqMan Universal Master Mix, no UNG (Applied Biosystems), TaqMan Probe (Thermo Fisher Scientific) and RNase‐free water. Standard PCR conditions were as follows: 10 minutes at 95°C (enzyme activation), followed by 60 cycles of 30 seconds at 95°C (denaturation), and 1 minute at 60°C (for annealing/extension). All samples were run in duplicate. Negative controls containing no cDNA were included in each RT‐qPCR run. The cycle threshold (*C*_t_) values were calculated automatically by a CFX96 Real‐Time PCR Detection System Software System (Bio‐Rad Laboratories Inc). For each sample, the gene expression of the target mRNA was calculated relative to a reference gene (Δ*C*_t sample_ = *C*_t target gene_ − *C*_t reference gene_). Levels of gene expression are given as a normalization ratio calculated as fold = 2^−ΔCt sample^. Fold change in the expression caused by venlafaxine administration was calculated using the 2 ^ (−ΔΔC_t_) method.^63^

### 2.6 Methylation and HRM analysis

The methylation status of gene promoters was specified by methylation‐sensitive high‐resolution melting.^64^, ^65^ Of the gene promotors studied in this assay, only the NOS2 promoter did not contain any CpG islands. In the case of genes expression regulated by many promoters, primers were designed for promoters containing CpG islands. Next, we designed primers using the Methyl Primer Express™ Software v 1.0 (Thermo Fisher Scientific). Bisulphite modification was performed using 200 ng of DNA with a CiTi Converter DNA Methylation Kit (A&A Biotechnology), according to the manufacturer's protocols. Methylated DNA (CpGenome™ Rat Methylated Genomic DNA Standard; Merck Millipore) and unmethylated DNA (CpGenome™ Rat Unmethylated Genomic DNA Standard; Merck Millipore), were used as controls for the MS‐HRM experiments; these were prepared following the same procedure as the blood and brain DNA samples.

In order to control for the sensitivity of methylation detection, serial dilutions were performed: non‐methylated, 10% methylated, 25% methylated, 50% methylated, 75% methylated and 100% methylated DNA (Figure S1). These methylation analyses were performed using the Bio‐Rad CFX96 Real‐Time PCR Detection System and analysed in HRM Powered by Precision Melt Analysis™ Software (Bio‐Rad Laboratories Inc). Each PCR contained 5× HOT FIREPol^®^ EvaGreen^®^ HRM Mix (no ROX) (Solis BioDyne), 500 nM of each primer and 10 ng of DNA after bisulphite modification (theoretical calculation) and was performed as three replicates. The parameters for amplification and HRM analyses included initial activation for 12 minutes at 95℃, 45 cycles of 95℃ for 15 seconds; annealing at optimal primer temperatures (tested experimentally) for 20 seconds (see Table 1 for characteristics of the primers), and elongation at 72℃ for 20 seconds. The HRM analysis consisted of denaturation at 95℃ for 15 seconds, reannealing at 60℃ for 1 minute and melting from 60 to 95℃ at a ramp rate of 0.2℃.

**TABLE 1:** The characteristics of primers used for analysis of methylation levels in the promoter regions of the studied genes

Gene	Starter sequence (5' ‐> 3')	Product size [bp]	Tm [^o^C]	Number of CpG islands in promotor region
CAT	F:TTTGAGATTATTGTGTTTGAAA R:TACCTACACCCAAAAAAAAATA	148	59	1
Gpx1	F:GTTGTTTTAGGTTTTGTTGTTG R:AAAACTAAAATCCTCCAACTCT	102	65	1
Gpx4 (promotor 2)	F:AGGTTGGAGGTTTAGAGGTTTA R:TCCCCTAAATACAAAAATCTCT	118	59	1
Gpx4 (promotor 3)	F:AGGTTGGAGGTTTAGAGGTTTA R:AAAACATAACAAAATCATCTCCC	147	65	1
SOD1	F: AAGGAGGTGTGTTTAATTGGTA R: AACCCCTCTCACAAATTTCTAA	144	65	1
SOD2	F: GGGGAAGGTTATTTAGGGTATA R: CCTTTTCCATTCCTAATTCTAAA	133	59	1
NOS1 (promotor 3)	F: GGGTTTTTAATTTTTTTATTGTG R: CAACCCTCATTAAAAAAACC	124	59	1
NOS1 (promotor 7)	F: GTTTGAGATTGGAATTTTTTGG R: CCAAAACATCCAAAAATACACA	124	59	1

### 2.7 Protein samples isolated from animal brains and the Western blot procedure

For the analysis, we selected proteins that are crucial for the regulation of oxidative stress, occurred in various cell locations (cytoplasm, mitochondria and cell nucleus) and have not been investigated in animal models of depression so far. The protein fractions isolated from the tissue of the rat brains were used for Western blotting.^98^ Frozen samples of the brain parts were homogenized using a FastGene^®^Tisue Grinder (Nippon Genetics Europe), in a RIPA buffer (10 mM Tris‐HCl PH 8.0, 1 mM EDTA, 1% Triton X‐100, 0.1% sodium deoxycholate, 0.1% SDS, 10 mM NaCl) containing 1 mM phenylmethylsulfonyl fluoride (PMSF, serine protease inhibitor). The homogenates were then sonicated twice for 15 seconds each in an ice bath and centrifuged at 2500 *g* for 5 minutes at 4℃. The supernatant was then collected. The protein content of the homogenate tissue fractions was estimated by means of the Lowry procedure,^71^ using bovine serum albumin (BSA). The samples (50 µg/lane) of homogenates were resolved with 10% polyacrylamide gel. Electrophoresis was conducted in a Tris/glycine/SDS buffer (25 mM Tris, 190 mM glycine, 0.2% SDS, pH 8.3), for 2.5 hours at 100 mA in OmniBlot Mini (Cleaver Scientific).

After electrophoresis, the samples were transferred onto a nitrocellulose membrane of Immobilon‐P (Millipore), in 25 mM Tris‐HCl containing glycine (190 Mm) and methanol (20%), and run at 55 V overnight.^99^ Then, the membranes were blocked with a blocking solution (5% non‐fat dry milk in a 0.1% TBST buffer—Tris buffered saline with Tween‐20) for 1 hour at room temperature. The blots were then incubated with primary antibodies: overnight at 4°C with mouse monoclonal antibodies specific to catalase (Santa Cruz Biotechnology Inc) and rabbit monoclonal antibodies specific to glutathione peroxidase 4 (Abcam). Primary mouse monoclonal antibodies specific to β‐actin (a reference protein) and superoxide dismutase 1 (Santa Cruz Biotechnology Inc) were incubated at room temperature for 1 and 2 hours, respectively. Dilutions of the antibodies were prepared according to the manufacturer's protocols. After being washed three times with 0.1% TBST, the membranes were incubated for 1 hour at room temperature with secondary goat anti‐rabbit or antimouse antibodies conjugated with horseradish peroxidase (Cell Signalling Technologies Inc), added in a 1:6000 dilution. Then, the blots were again washed six times with 0.1% TBST and incubated with peroxidase substrate solution. For chemiluminescent reaction, the membranes were incubated for 3 minutes in a stable peroxide solution, an enhanced luminol solution and water in a 1:1:1 proportion (Thermo Fisher Scientific). The proteins were visualized on X‐ray film by enhanced chemiluminescence. Using Gel‐Pro^®^ Analyzer Software (Media Cybernetics Inc), the integrated optical density (IOD) of the immunoreactivity bands was measured from digital images. The level of protein expression was normalized using the reference protein—beta‐actin (ACTB; IOD_gene/_IOD_ACTB_).

### 2.8 Drugs

Venlafaxine HCl (Carbosynth Ltd) was dissolved in 0.9% sterile saline, which was used for vehicle administration, and administered IP in a volume of 1 mL/kg of body weight, at the dose of 10 mg/kg, as used previously.^61^, ^62^

### 2.9 Statistical analysis

The effect of initial 2 weeks of CMS on sucrose consumption was analysed by test *t* when the data were normally distributed or Mann‐Whitney rank‐sum Test when the data were not normally distributed. The sucrose intakes, gene expression and methylation data were analysed using an one‐way analysis of variance (one‐way ANOVA), followed by post hoc Tukey's test, where *F* ratios were significant for the following groups: Controls/Vehicle, Stressed/Vehicle and Stressed/Venlafaxine when the data were normally distributed. Otherwise, the data were analysed using a Kruskal‐Wallis one‐way analysis of variance on ranks followed by post hoc Student‐Newman‐Keuls test, where *H* ratios were significant for the studied groups. Student's *t* test was used to analyse differences between blood and brain samples. *P* values <.05 were considered significant. Statistics were calculated using Statistica 12 (StatSoft), SigmaPlot 11.0 (Systat Software Inc) and GraphPad Prism 5.0 (GraphPad Software, Inc).

## 3 RESULTS

### 3.1 Sucrose intakes and body weights of animals exposed to CMS and venlafaxine

As shown in Table 2, before the stress was initiated (Week 0), the consumption of 1% sucrose solution was comparable in all groups. Following initial 2 weeks of stress (Week 2), the intakes decreased by approximately 40% (*F* = 6.76, fd = 2, *P* < .01, Tukey's test *P* = .01) and remained low until the end of experiment (Week 7) in stressed animals treated with vehicle. Chronic (5 weeks) administration of venlafaxine had no effect in control animals but normalized the intakes in stressed rats (*F* = 6.76, fd = 2, *P* < .01, Tukey's test *P* = .01). Neither stress nor venlafaxine had any significant effect on body weights of control or stressed animals (data not shown).

**TABLE 2:** Sucrose intakes in animals exposed to chronic mild stress (CMS) for 2 wk (Week 2) and in animals exposed to CMS for 7 wk (Week 7) and administered vehicle (1 mL/kg) or venlafaxine (10 mg/kg) for 5 wk

Weeks of CMS	Control	Stressed	Control/Venla	Stressed/Saline	Stressed/Venla
Week 0	12.6 ± 1.6	11.0 ± 0.7	11.7 ± 0.7	11.9 ± 0.7	11.4 ± 0.5
Week 2	15.6 ± 1.9	6.8 ± 1.0^##^	4.9 ± 0.6^@@@^	13.9 ± 0.9	5.8 ± 0.5^&^
Week 7			6.1 ± 0.7	13.3 ± 1.3	12.6 ± 1.0**

The data represent means ± SEM. N = 6.

^##^ *P* < .01; relative to Week 2 in the Stressed group.

^&^ *P* < .05; relative to Week 2 in the Stressed/Venla group.

** *P* < .01; relative to Week 2 in the Stressed/Venla group.

^@@@^ *P* < .001; relative to Week 0 in the Control/Venlafaxine group.

### 3.2 Gene expression at the mRNA level

#### 3.2.1 Gene expression in PBMCs

**FIGURE 1:**
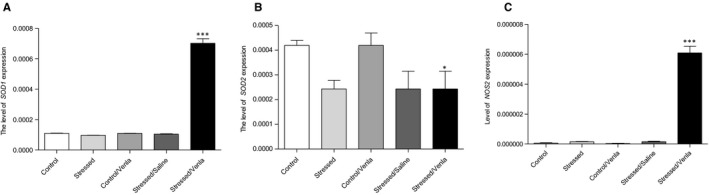
mRNA expression of *SOD1* (A), *SOD2* (B) and *NOS2* (C) genes in PBMCs of animals exposed to chronic mild stress (CMS) for 2 wk (Control, Stressed) and in animals exposed to CMS for 7 wk and administered vehicle (1 mL/kg) or venlafaxine (10 mg/kg) for 5 wk (Control/Venla, Stressed/Saline, Stressed/Venla). Relative gene expression levels were estimated using a 2^−ΔCt (Ctgene–Ct18S)^ method. Data represent means ± SEM. N = 6. *P < .05; ***P < .001; relative to Stressed/Saline group

The expression of *Gpx1*, *Gpx4* and *CAT* mRNA in PBMCs did not differ between the two groups exposed to the control and stress conditions for 2 weeks (Table S2), as well as between the control and stressed animals treated with saline (Figure 1.). Chronic (5 weeks) administration of venlafaxine had no effect in control animals but significantly increased the expression of *SOD1* (*F* = 6.49, *df* = 2, *P* < .001, Tukey's test *P* < .001), *SOD2* (*F* = 7.20, *df* = 2, *P* < .01, Tukey's test *P* < .05) and *NOS2* (*F* = 6.49, *df* = 2, *P* < .001) Tukey's test *P* < .001) genes in the stressed rats after venlafaxine therapy.

#### 3.2.2 Gene expression in brain

**FIGURE 2:**
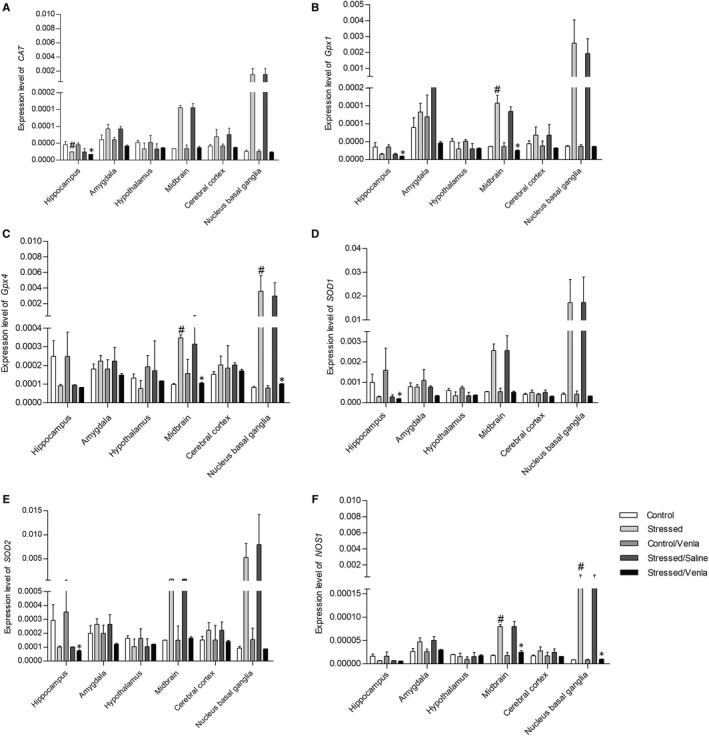
mRNA expression of *CAT*, *Gpx1*, *Gpx4*, *SOD1*, *SOD2* and *NOS1* in brain structures of animals exposed to chronic mild stress (CMS) for 2 wk (Control, Stressed) and in animals exposed to CMS for 7 wk and administered vehicle (1 mL/kg) or venlafaxine (10 mg/kg) for 5 wk (Control/Venla, Stressed/Saline, Stressed/Venla). Relative gene expression levels were estimated using a 2^−ΔCt (Ctgene–Ct18S)^ method. Data represent means ± SEM. N = 6. *P < .05; relative to Stressed/Saline group. ^#^ *P* < .05; relative to Control group

As shown in Figure 2, the effect of CMS and venlafaxine on the mRNA expression of the studied genes was clearly dependent on the structure, while CMS decreased the hippocampal expression of *CAT* (*F* = 7.20, *df* = 2, *P* < .01, Tukey's test *P* < .05), which was further down‐regulated after venlafaxine administration (*P* < .05). On the other hand, the procedure increased the expression of *Gpx1* in midbrain (*F* = 7.20, *df* = 2, *P* < .01, Tukey's test *P* < .05), and *Gpx4* and *NOS1* expression in both midbrain and nucleus basal ganglia, all of which returned to control levels after administration (*F* = 7.20, *df* = 2, *P* < .01, Tukey's test *P* < .05). Moreover, after treatment with venlafaxine, the stressed animals were characterized by down‐regulated hippocampal expression of *SOD1* (*F* = 6.49, *df* = 2, *P* < .05, Tukey's test *P* < .05), *SOD2* (*F* = 5.96, *df* = 2, *P* < .05, Tukey's test *P* < .05) and Gpx1 (*F* = 5.96, *df* = 2, *P* < .05, Tukey's test *P* < .05). Additionally, no differences in mRNA expression were found between the non‐stressed group and the control animals after venlafaxine administration, or between the CMS group and the CMS group after vehicle administration.

#### 3.2.3 Comparison of gene expressions in PMBCs and brain

**FIGURE 3:**
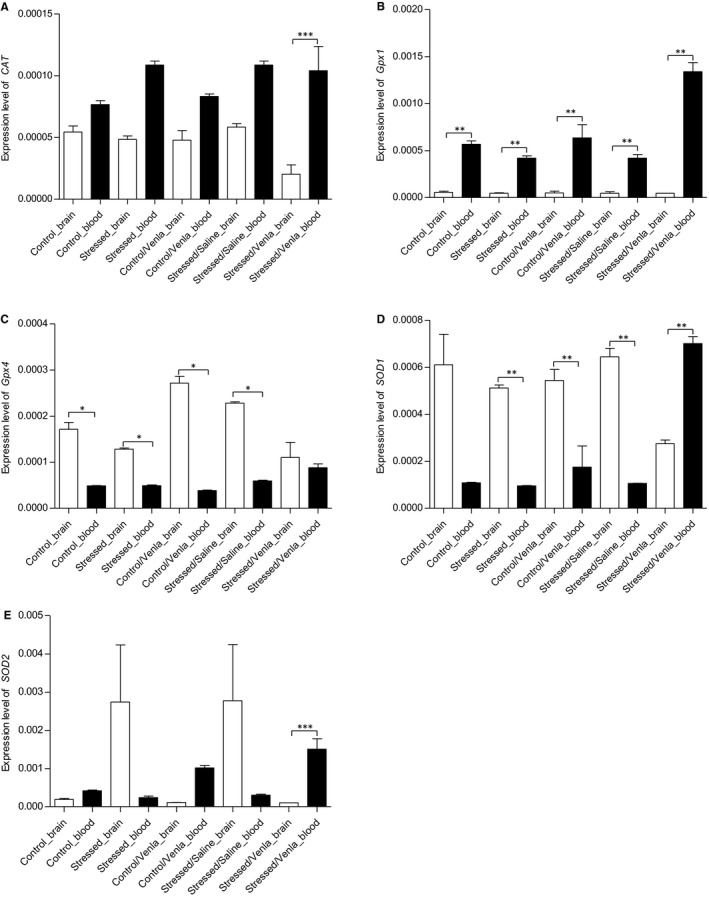
Differences in *CAT* (A), *Gpx1* (B), *Gpx4* (C), *SOD1* (D) and *SOD2* (E) gene expression between brain tissue and PBMCs of animals exposed to chronic mild stress (CMS) for 2 wk (Control, Stressed) and in animals exposed to CMS for 7 wk and administered vehicle (1 mL/kg) or venlafaxine (10 mg/kg) for 5 wk (Control/Venla, Stressed/Saline, Stressed/Venla). The mRNA relative expression levels of all five genes measured in hippocampus, amygdala, hypothalamus, midbrain, cortex and basal ganglia were pooled and compared with the data detected in PBMCs. Relative gene expression levels were estimated using a 2^−ΔCt (Ct gene–Ct 18S)^ method. Data represent means ± SEM. N = 6. *P < .05, **P < .01, ***P < .001

In order to compare effects of CMS and venlafaxine on the expression of *CAT*, *Gpx1*, *Gpx4*, *SOD1*, *SOD2* and *NOS1* genes in the brain and in blood, all data measured in the brain structures were pooled and compared with the changes detected in PMBCs. As shown in Figure 3, in all groups the expression of Gpx1 was higher, and the expression of *GPx4* lower, in the PBMCs than in the brain structures (*P* < .01 and *P* < .05, respectively). The expression of *CAT* and *SOD2* were lower in the brain tissue of the stressed rats treated with venlafaxine (*P* < .001). In addition, the expression of *SOD1* in brain tissue was higher than that observed in PBMCs of the stressed rats and the stressed rats treated with saline and venlafaxine (*P* < .01).

#### 3.2.4 Effect of venlafaxine on the expression of genes in PMBCs and brain structures

**FIGURE 4:**
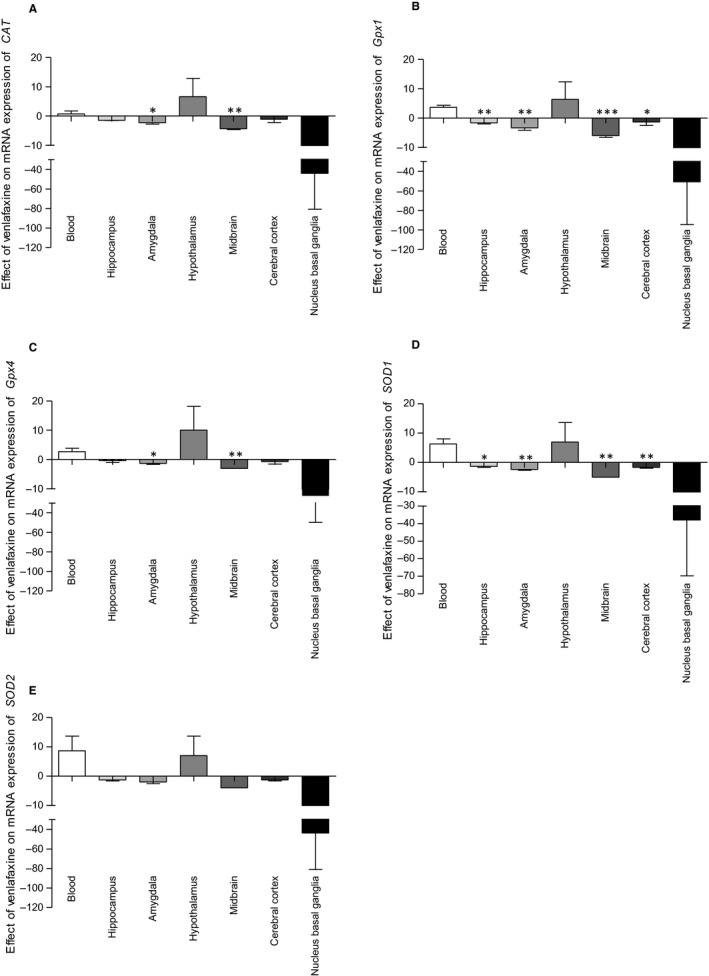
mRNA expression of *CAT* (A), *Gpx1* (B), *Gpx4* (C), *SOD1* (D) and *SOD2* (E) in PBMCs and in brain structures of animals exposed to chronic mild stress (CMS) for 2 wk (Control, Stressed) and in animals exposed to CMS for 7 wk and administered vehicle (1 mL/kg) or venlafaxine (10 mg/kg) for 5 wk (Control/Venla, Stressed/Saline, Stressed/Venla). The effects are presented as fold change (2^−ΔΔCt^ method^51^). Data represent means ± SEM. N = 6. *P < .05, **P < .01, ***P < .001; relative to PBMCs

Venlafaxine significantly decreased the expression of *CAT* and *Gpx4* in amygdala (*P* < .05) and midbrain (*P* < .01), when compared to the expression of these genes in the PMBCs (Figure 4). Administration of venlafaxine caused a down‐regulation of *Gpx1* and *SOD1* expression (Figure 4) in hippocampus (*P* < .01, *P* < .05, respectively), amygdala (*P* < .01), midbrain (*P* < .001, *P* < .01, respectively) and cerebral cortex (*P* < .05, *P* < .01, respectively), as compared to its effects in PBMCs. No differences in the effect of venlafaxine on SOD2 expression was found between the blood and brain tissue.

### 3.3 Methylation of the studied gene promoters

#### 3.3.1 Methylation in PBMCs

**FIGURE 5:**
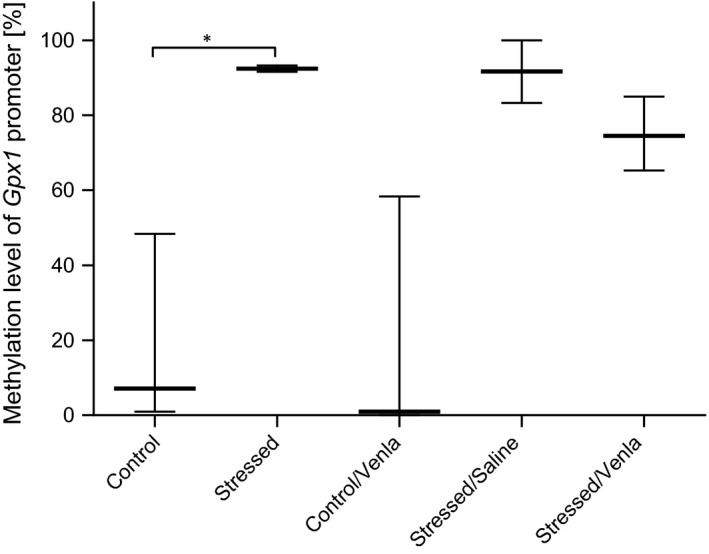
Methylation level of *Gpx1* promoter in PBMCs of animals exposed to chronic mild stress (CMS) for 2 wk (Control, Stressed) and in animals exposed to CMS for 7 wk and administered vehicle (1 mL/kg) or venlafaxine (10 mg/kg) for 5 wk (Control/Venla, Stressed/Saline, Stressed/Venla). Data represent median and maximum‐minimum values. N = 6. *P < .05

The only significant change that was found in PBMCs was an increased methylation of *Gpx1* promoter region in animals exposed to the CMS procedure for 2 weeks (*H* = 12.83, *df* = 2, *P* < .05, Tukey's test *P* < .05; Figure 5).

No significant differences between groups were observed for promoters of other genes in PBMCs (Table S3).

#### 3.3.2 Methylation in brain

**FIGURE 6:**
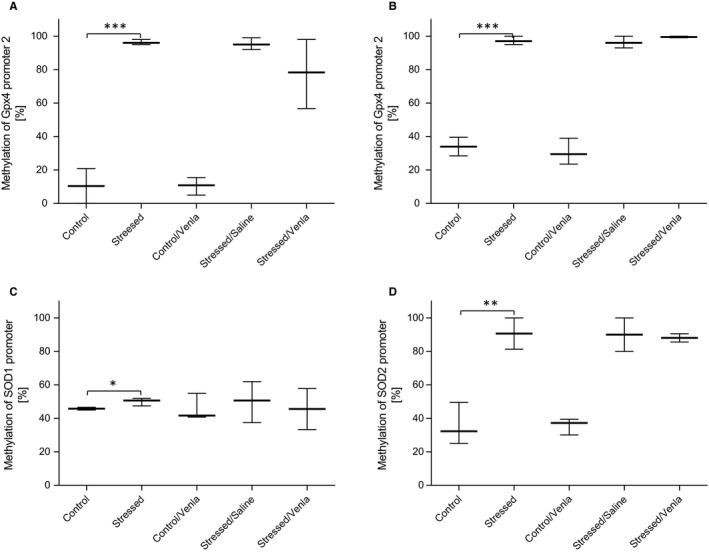
Methylation levels of *Gpx4* promoter 2 in midbrain (A) and basal ganglia (B), *SOD1* (C) and *SOD2* (D) promoter in hippocampus of animals exposed to chronic mild stress (CMS) for 2 wk (Control, Stressed) and in animals exposed to CMS for 7 wk and administered vehicle (1 mL/kg) or venlafaxine (10 mg/kg) for 5 wk (Control/Venla, Stressed/Saline, Stressed/Venla). Data represent means ± SEM (A) or median and minimum‐maximum values (B–D). N = 6. *P < .05, **P < .01, ***P < .001

As shown in Figure 6, the CMS procedure significantly increased methylation level of the second promoter of *Gpx4* in midbrain (*F* = 28.19, *df* = 2, *P* < .001, Tukey's test *P* < .001) and basal ganglia (*H* = 6.76, *df* = 2, *P* < .05, Tukey's test *P* < .05) and methylation levels of *SOD1* and *SOD2* promoters in hippocampus (*H* = 5.14, *df* = 2, *P* < .05, Tukey's test *P* < .05; *H* = 5.96, *df* = 2, *P* < .05, Tukey's test *P* < .01, respectively). No other significant differences between groups were observed for the other promoters in the brain structures (Table S4).

#### 3.3.3 Comparison of the methylation status of gene promoters in PBMCs and brain

**FIGURE 7:**
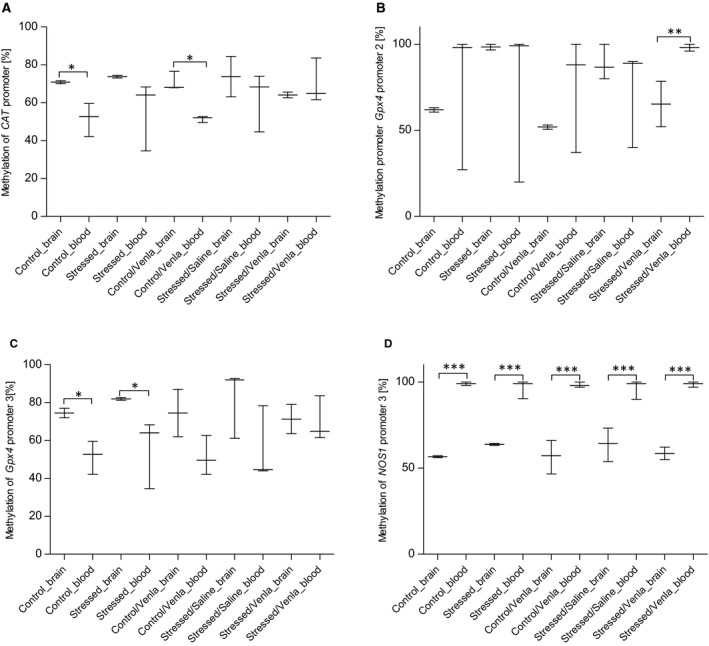
Differences in methylation level of *CAT* (A), *Gpx4* promoter 2 (B), *Gpx4* promoter 3 (C) and *NOS1* promoter 3 (D) between brain tissue and PBMCs of animals exposed to chronic mild stress (CMS) for 2 wk (Control, Stressed) and in animals exposed to CMS for 7 wk and administered vehicle (1 mL/kg) or venlafaxine (10 mg/kg) for 5 weeks (Control/Venla, Stressed/Saline, Stressed/Venla). The methylation level of all four genes measured in hippocampus, amygdala, hypothalamus, midbrain, cortex and basal ganglia were pooled and compared with the data detected in PBMCs. Data represent median and maximum‐minimum values. N = 6. *P < .05, **P < .01, ***P < .001

In order to compare the effects of CMS and venlafaxine on the brain and peripheral expression of *CAT*, *Gpx1*, *Gpx4*, *SOD1*, *SOD2* and *NOS1* genes, all results obtained for the brain structures were pooled and compared with changes detected in the PBMCs (Figure 7).

The untreated and venlafaxine‐treated control animals showed an increased methylation in the promoter region of *CAT* and the third *Gpx4* promoter in brain tissue, compared to PBMCs (*P* < .05). In the stressed animals treated with venlafaxine, the methylation level of the second *Gpx4* promoter was lower in the brain than in PBMCs (*P* < .01). In all groups, the third promoter of NOS1 demonstrated lower methylation status in brain tissue than in PBMCs (*P* < .001). No other significant differences were observed for other promoters (Table S5).

#### 3.3.4 Effect of venlafaxine on the methylation status of gene promoters in PBMCs and brain structures

**FIGURE 8:**
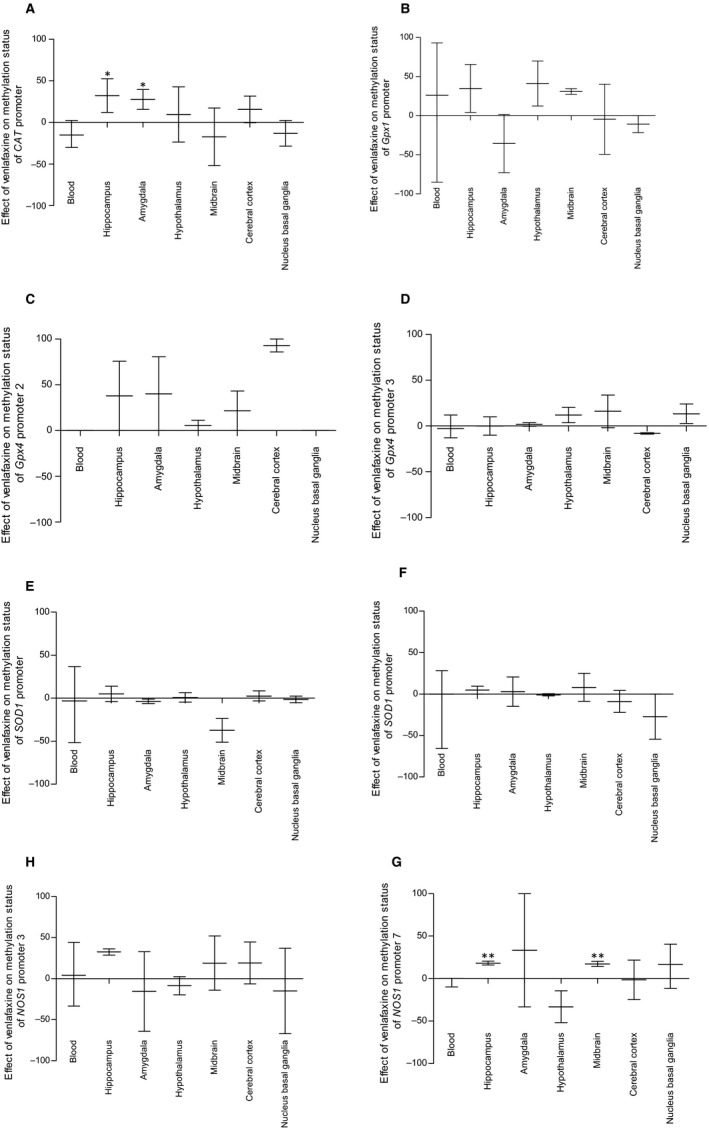
Differences in methylation level of *CAT* (A), *Gpx1* (B), *Gpx4* promoter 2 (C), *Gpx4* promoter 3 (D), *SOD1* (E), *SOD2* (F), *NOS1* promoter 3 (G) and *NOS1* promoter 7 (H) between brain tissue and PBMCs of animals exposed to chronic mild stress (CMS) for 2 wk (Control, Stressed) and in animals exposed to CMS for 7 wk and administered vehicle (1 mL/kg) or venlafaxine (10 mg/kg) for 5 wk (Control/Venla, Stressed/Saline, Stressed/Venla). The methylation level of all eight genes measured in hippocampus, amygdala, hypothalamus, midbrain, cortex and basal ganglia were pooled and compared with the data detected in PBMCs. Data represent means ± SEM. N = 6. *P < .05, **P < .01; relative to PBMCs

Compared to the effect of venlafaxine in PBMCs, the drug caused a stronger increase in the methylation level of *CAT* promoter in amygdala and hippocampus (*P* < .05) and the NOS1 seventh promoter in hippocampus and midbrain (*P* < .01) (Figure 8).

### 3.4 Gene expression on the protein level

**FIGURE 9:**
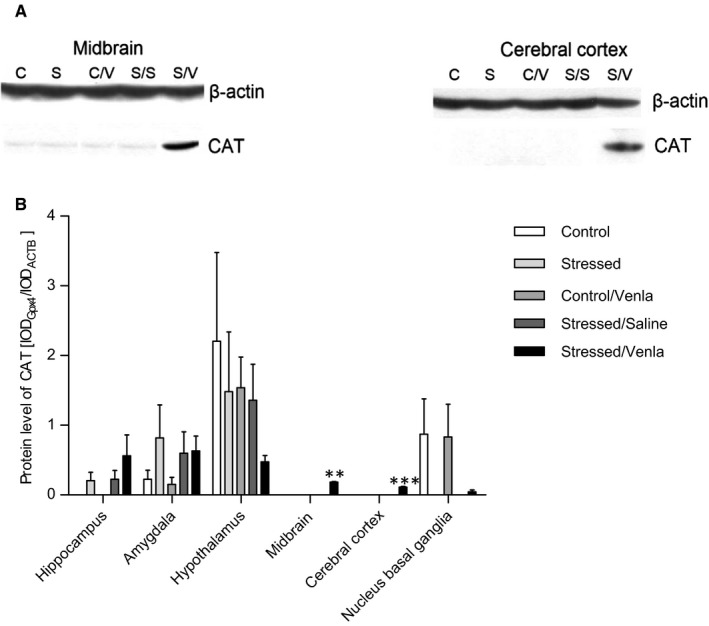
mRNA expression of CAT in brain structures of animals exposed to chronic mild stress (CMS) for 2 wk (Control, Stressed) and in animals exposed to CMS for 7 wk and administered vehicle (1 mL/kg) or venlafaxine (10 mg/kg) for 5 wk (Control/Venla, Stressed/Saline, Stressed/Venla). A, Representative Western blot analysis of the effects in midbrain and cerebral cortex. C—controls, S—stressed for 2 wk, C/V—Control/Venlafaxine, S/S—Stressed/Saline, S/V—Stressed/Venlafaxine. B, Level of CAT proteins measured in hippocampus, amygdala, hypothalamus, midbrain, cortex and basal ganglia. Samples containing 25 μg of proteins were resolved by SDS‐PAGE. The intensity of bands corresponding to CAT was analysed by densitometry. Integrated optical density (IOD) was normalized by protein content and a reference sample (see the Methods section for details). The graphs show the mean IODs of the bands from all analysed samples. The IOD_gene_/IOD_ACTB_ method was used to estimate the relative protein expression levels in the analysed samples. Data represent means ± SEM. N = 6. **P < .01, ***P < .001; relative to Stressed/Saline group

**FIGURE 10:**
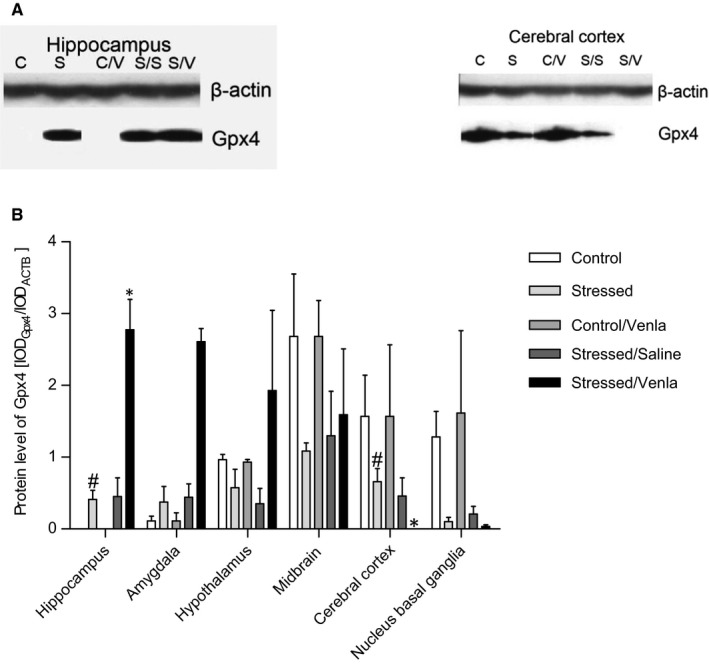
mRNA expression of Gpx4 in brain structures of animals exposed to chronic mild stress (CMS) for 2 wk (Control, Stressed) and in animals exposed to CMS for 7 wk and administered vehicle (1 mL/kg) or venlafaxine (10 mg/kg) for 5 wk (Control/Venla, Stressed/Saline, Stressed/Venla). A, Representative Western blot analysis of the effects in hippocampus and cerebral cortex. C—controls, S—stressed for 2 wk, C/V—Control/Venlafaxine, S/S—Stressed/Saline, S/V—Stressed/Venlafaxine. B, Level of Gpx4 proteins measured in hippocampus, amygdala, hypothalamus, midbrain, cortex and basal ganglia. Samples containing 25 μg of proteins were resolved by SDS‐PAGE. The intensity of bands corresponding to CAT was analysed by densitometry. Integrated optical density (IOD) was normalized by protein content and a reference sample (see the Methods for details). The graphs show the mean IODs of the bands from all analysed samples. The IOD_gene_/IOD_ACTB_ method was used to estimate the relative protein expression levels in the analysed samples. Data represent means ± SEM. N = 6. **P < .01, ***P < .001; relative to Stressed/Saline group. **P < .01, ***P < .001; relative to Stressed/Saline group. ^#^ *P* < .05; relative to Control group

As shown in Figure 9, venlafaxine significantly elevated the level of CAT protein in midbrain and cerebral cortex (*F* = 7.62, *df* = 2, *P* < .01, Tukey's test *P* < .01; *F* = 15.22, *df* = 2, *P* < .001, Tukey's test *P* < .001, respectively). The level of Gpx4 protein was elevated in hippocampus of the stressed animals (*F* = 7.45, *df* = 2, *P* < .01, Tukey's test *P* < .05) (Figure 10), and this effect was further enhanced following venlafaxine treatment (*F* = 7.45, *df* = 2, *P* < .01, *P* < .05). In cerebral cortex, the Gpx4 protein level was decreased in stressed animals (*F* = 6.16, *df* = 2, *P* < .05, Tukey's test *P* < .05), and again, this effect was enhanced by venlafaxine treatment (*F* = 6.16, *df* = 2, *P* < .05, Tukey's test, *P* < .05). No significant effects were observed in the other proteins (Table S6).

## 4 DISCUSSION

The present study is the first to investigate effects of repeated administration of venlafaxine on the expression of selected genes in the PBMCs of rats exposed to the CMS model of depression. Our results also demonstrate the CMS‐ and venlafaxine‐induced changes in the genes involved in oxidative and nitrosative stress in PBMCs and in six regions of the brain (hippocampus, amygdala, hypothalamus, midbrain, cerebral cortex and basal ganglia) with regard to their promoter methylation status and their expression of the mRNA and protein levels. In this study, genes expression were measured, rather than, activity of the antioxidant enzymes to highlight the role of epigenetic changes, that is promoter methylation, which is possible only when DNA, RNA and proteins are isolated from the same sample. There are reports showing that the expression of antioxidant enzymes is correlated with their activity^66^, ^67^ so the proteins selected in this study were not enzymatically active and, therefore, could only be quantified using either Western blot or immunosorbent assays.

The first studied gene encodes catalase (CAT), an important antioxidative enzyme that decomposes hydrogen peroxide into water and oxygen.^68^ It is believed to be involved in the mechanisms of depression but the exact nature of this involvement remains unclear. Our findings indicate that *CAT* expression in hippocampus was lowered in animals exposed to the CMS procedure, and this finding is in line with other studies showing that chronic stress causes a reduction in cortical and hippocampal catalase activity.^34^, ^35^ Similar reductions in enzyme activity were observed in the erythrocytes of depressed patients.^69^ However, CAT activity was also found to be increased in the serum of these patients,^70^ a finding inconsistent with our observation that the CMS procedure did not affect *CAT* expression in PBMCs. Interestingly, we found that administration of venlafaxine to stressed animals significantly elevated the level of CAT protein in their midbrain and cerebral cortex. Similarly, catalase activity increased in the nucleus accumbens of rat brains after administration of quetiapine, an atypical antipsychotic used also in the pharmacotherapy of depression.^36^ In addition, a study employing a model of behavioural despair in mice showed that venlafaxine restored reduced CAT activity in the brain.^41^ However, our present findings do not suggest that the peripheral expression of CAT was affected by venlafaxine, which is in line with report by Ozcan et al^69^ that the treatment with venlafaxine did not change the CAT activity in erythrocytes.

Another antioxidant enzyme believed to be associated with mechanisms of depression is GSH peroxidase (Gpx), which reduces lipid hydroperoxides and free hydrogen peroxide.^72^ Gpx1 and Gpx4 have been shown to be selenium‐containing enzymes. While Gpx1 is present only in the cytosol, Gpx4 is located in the membrane and mitochondria.^73^ Our findings indicate that the mRNA expression of Gpx1 was increased in midbrain of the CMS rats. In contrast, Eren et al^74^ found that Gpx activity in the cortex is lowered in rats subjected to the CMS procedure and that venlafaxine administration normalized this effect. In humans, Gpx1 activity was found to be decreased in the haemolysed erythrocytes of depressed women ^32^ and increased in the erythrocytes of patients with melancholia.^75^ Moreover, the changes in the Gpx1 concentration have been found to be negatively correlated with the severity of depressive symptoms.^76^ Our present findings indicate that the midbrain and hippocampal expression of *Gpx1* mRNA was significantly lower in the venlafaxine‐treated stressed animals than in the vehicle‐treated stressed rats. Accordingly, the activity of Gpx in serum was found to decrease over the course of 3‐month administration of different SSRIs.^75^ In the present study, higher *Gpx1* mRNA expression was observed in PBMCs than in brain tissue in all of the stress and treatment conditions, and the methylation of the Gpx1 promoter in PBMCs was lower in the control than the CMS rats: a key novel finding of other crucial regulation of mRNA gene expression.

This study is also the first to examine the changes in the level of Gpx4 isoform in an animal model of depression. The CMS procedure increased the expression of Gpx4 mRNA in midbrain and basal ganglia, and this effect was normalized by administration of venlafaxine. Interestingly, the methylation levels of the second Gpx4 promoter in these two brain regions were also higher in the stressed group. This however did not affect the level of mRNA expression, because the *Gpx4* gene contains six promoters and only one of them was found to have higher methylation status in animals exposed to the CMS procedure. CMS also increased the Gpx4 protein level in hippocampus, and venlafaxine caused a further increase in Gpx4 protein expression while the opposite trend was observed in cerebral cortex. The differences between the expression of *Gpx4* mRNA and protein could be a result of epigenetic or posttranscriptional modifications, for example the influence of microRNA. Moreover, the presence of a high level of *Gpx4* mRNA expression and a high methylation status of the *Gpx4* promoter could suggest that gene expression is subject to other forms of regulation than the methylation of promoter regions. Other epigenetic modifications that alter DNA accessibility and chromatin structure, thereby regulating patterns of gene expression, could also be considered, that is histone modification (such as methylation and acetylation) and nucleosome positioning. These processes are crucial to the normal development and functioning of cells.^77^ The results may also indicate the involvement of other, post‐transcriptional epigenetic regulation, that is RNA silencing by microRNA, as the mRNA expression of the studied genes was found to differ from their respective protein levels. As such, the role of these processes should be addressed in future studies.

The next antioxidant enzyme associated with the mechanism of depression is SOD. In post‐mortem clinical studies, increases in SOD1 and SOD2 levels were found in frontal cortex of patients with schizophrenia and in prefrontal cortex of depressive subjects.^78^ A greater erythrocyte activity of SOD1 was also found in patients with depression^75^; however, there are reports showing opposite effects: lower serum^31^, ^37^ and erythrocyte SOD1 activity^79^ in depressed patients. In the present study, we did not observe any significant effect of the CMS procedure on the PBMCs level of SOD1 protein.

The concentration of SOD1 in serum of depressed patients is decreased during the course of citalopram and fluoxetine therapy^24^; this finding is consistent with our observation of increased *SOD1* mRNA level in hippocampus of stressed animals administered venlafaxine. Similarly, Galecki et al^80^ reported decreased SOD1 activity in the red blood cells (RBCs) of patients treated with combined fluoxetine and acetylsalicylic acid therapy; however, our present findings indicate that SOD1 mRNA expression in PBMCs of the CMS rats was increased by venlafaxine. *SOD2* mRNA expression and protein activity is lower in depressed patients,^81^ and the RBCs activity of SOD2 is positively associated with severity of the disease.^82^ Our present animal data show that *SOD2* mRNA expression in PBMCs was greater in CMS rats after venlafaxine therapy compared to the CMS group. Similarly, while SOD2 activity was previously found to be elevated in brain tissue of animals treated with venlafaxine,^59^ our findings indicate that hippocampal *SOD2* mRNA expression is decreased after venlafaxine therapy. There is evidence that overexpression of *SOD2* could play a crucial role in reducing oxidative stress and preventing neurodegenerative disease^83^ and that reduced *SOD2* expression in the cerebral cortex, cerebellum and basal ganglia may lead to neurodegeneration.^84^ The differences between blood and brain in rats after therapy may be result of different tissue response to treatment. Probably, enzyme activity might have been more susceptible to antidepressant therapy in blood than in the brain. Moreover, these changes in SOD2 concentration may be responsible for the reductions in the volume of prefrontal cortex and hippocampus that are characteristic of patients with depression.^85^ Therefore, the reduction in brain structure volume may cause irreversible changes in antioxidant capacity, resulting in no response to treatment in brain. Our present results show that the *SOD2* promoter region displayed higher methylation status in the stressed groups, suggesting that the antioxidant defence in the stressed animals was indeed less efficient. It should be however noted that only one of the two *SOD2* promoters was characterized by high methylation status, and no differences in SOD2 protein level were observed.

NOS generates NO, which plays a key role in many biological processes and in regulation of cognitive and emotional functions. Overproduction of NO can lead to oxidative and antioxidative imbalance, which is believed to be involved in pathomechanisms of neurodegenerative diseases, including anxiety and depression.^86^, ^87^ Previous studies confirmed that depressed patients are characterized by an increased level of NO in plasma.^88^, ^89^ In line with this, the CMS rats and mice showed an increased level of *NO* in cortex.^74^, ^90^ Our results also indicate that the *NOS1* mRNA expression in midbrain and basal ganglia was higher in rats exposed to the CMS procedure. However, no changes were observed in the stressed animals administered venlafaxine, which is inconsistent with the results obtained by Yoshino et al^50^ who reported that antidepressant treatment increased *nNOS* (*NOS1*) mRNA expression in the hippocampus, midbrain, cerebellum and olfactory bulb,^50^ *iNOS* (*NOS2*) mRNA expression in the frontal cortex and midbrain, and decreased *eNOS* mRNA expression in most brain regions. Interestingly, in the study which used a similar CMS paradigm, Wang et al reported that the suppression of hippocampal NOS can protect against the development of depressive‐like symptoms in this model of depression.^92^

The presence of elevated NO levels in depressed patients could be a result of an increased expression of cellular *NOS1* in suprachiasmatic nucleus, cornu ammonis area 1 and subiculum regions.^92^ The serum NO level was significantly decreased in depressed patients after 8 weeks of SSRI treatment,^37^ while an earlier animal study indicated that CMS caused a deformation of neurons in the hippocampus, while fluoxetine therapy led tonormalization of these neurons by inhibiting NOS1.^93^ Hence, it is likely that CMS could cause a nitrosative stress that is inhibited by venlafaxine‐induced reduction in NO generation. However, the level of NO in cortex of the CMS animals was found to be reduced following venlafaxine treatment.^85^ In the present study, no significant differences were detected between any groups with regard to NOS1 protein level in the brain. Talarowska et al (2015) found no significant difference in NOS2 levels between patients with recurrent depressive disorders and those in a first episode of depression.^95^ However, our findings indicate that in the CMS animals venlafaxine did increase mRNA expression of NOS2 in PBMCs compared to the untreated stressed group.

This study is the first to report the effect of CMS and antidepressant treatment on the expression and methylation status of genes involved in oxidative and nitrosative stress in both the blood and the brain in the same experimental setting. Such comparative analysis is not possible to carry out in humans, because it would require too many subjects, and the brain tissue would be available post‐mortem only. Hence, the use of a validated animal model of depression, which can pinpoint most promising genes that can be then verified in depressed patients, seems to be most reasonable for identification peripheral markers of the central nervous system pathology. We found that the mRNA expression of *Gpx1* was significantly higher, and that of *Gpx4* significantly lower, in PBMCs than the brain structures in all groups. Also, the expression of SOD1 mRNA was higher in the brains of all control and stressed groups, except for the stressed animals successfully treated with venlafaxine, which showed higher expression of this gene in the PBMCs. Similar enhanced expression in the PBMCs of the venlafaxine‐treated stressed animals was observed for the *CAT* and *SOD2* genes; however, in other groups these genes either did not change (*CAT*) or changed regardless of the conditions (*SOD2*).

The present findings and those of previously reported by others ^28^, ^29^, ^30^, ^31^, ^50^, ^95^ suggest that the lack of sufficient enzymatic antioxidant defence in PBMCs and brain are involved in effects observed in animals exposed to the CMS procedure, a conclusion that could be cautiously extrapolated to the mechanisms of human depression. We can speculate that this inadequate antioxidant defence leads to increased production of toxic free radicals which, in excessive amounts, imply damage to biomolecules. In consequence, prolonged exposure to free radicals may cause a cell death, neuronal and glia atrophy leading to decreased grey matter volume, reduced cell numbers and low glucose metabolism observed in depressed patients.^96^, ^97^ On the other hand, antidepressant therapy may modulate the activity of enzymes restoring this antioxidant defence system to its proper functioning. Accordingly, our findings confirm that venlafaxine reduces the mRNA expression of the genes encoding the SOD1, SOD2, Gpx1, Gpx4, CAT and NOS1 enzymes in the brain, but increases that of SOD1, SOD2 and NOS2 in PBMCs. Only brain expression of *Gpx1*, *Gpx4* and* NOS1* mRNA was reduced to control levels by venlafaxine therapy. The decrease in the level of antioxidant enzyme gene expression may indicate reduced production of free radicals after venlafaxine therapy, and the decreased expression of genes involved in oxidative stress may, in turn, indicate effectiveness of the antidepressant treatment.

Unfortunately, our results were characterized by a large variability. Depression is a heterogeneous disease and the clinical symptoms vary among patients. The distinct symptoms may be either present or absent in individual cases of depression, or even go in opposite directions. The opposing symptoms are also seen in patients with depression—excessive or lack of appetite, insomnia or excessive sleepiness Thus, the developing a stable model of this disease is very difficult. Moreover, a many symptoms of human depression, including feelings of worthlessness, guilt, or thoughts of death, cannot be modelled in animals. The animal species, strain and sex are also important in preparation of a valid animal model of depression. Additionally, the disease genes for depression are unknown, and thus, many transgenic models and models based on behavioural selection might not represent valid models of the disease.^11^

## 5 CONCLUSION

Our findings indicate that oxidative and nitrosative stress are involved in the effects of CMS and venlafaxine administration, more specifically, (a) the CMS procedure changed the expression of *CAT*, *Gpx1*, *Gpx4* and *NOS1* at the mRNA level only in the brain; (b) chronic administration of venlafaxine affected the mRNA expression of *SOD1*, *SOD2* and *NOS1* both in the PBMCs and in the brain, and *CAT*, *Gpx1* and *Gpx4* only in the brain; (c) changes in promoter methylation was caused only by the CMS procedure in both the PBMCs and the brain; (d) contradictory results were found concerning the influence of promoter methylation on mRNA expression, that is an increased methylation status of the *Gpx4* promoter was associated with increased expression, showing that other epigenetic factors, like histone modifications, can affect the expression of the studied genes; and (e) the results obtained using peripheral tissue could predict the condition of the brain; however, this was dependent on brain structure. These findings provide strong evidence for thesis that analysis of the level of mRNA and protein expression as well as the status of promoter methylation can help in understanding the pathomechanisms of mental diseases, including depression, and the mechanisms of action of drugs effective in their therapy.

## CONFLICT OF INTEREST

The authors declare no conflicts of interest.

## AUTHOR CONTRIBUTIONS

Tomasz Śliwiński and Mariusz Papp conceived and planned the experiments; Paulina Wigner, Ewelina Synowiec, Piotr Czarny and Piotr Gruca contributed to sample preparation; Paulina Wigner, Ewelina Synowiec, Piotr Czarny, Michal Bijak, Paweł Jóźwiak, Piotr Gruca and Janusz Szemraj carried out the experiment; Paulina Wigner, Ewelina Synowiec, Mariusz Papp and Tomasz Śliwiński analysed the data; Paulina Wigner, Mariusz Papp and Tomasz Śliwiński wrote the manuscript and designed the figures and tables. All authors provided critical feedback and helped shape the research, analysis and manuscript.

## Supporting information

Supplementary Material

## Data Availability

The data that support the findings of this study are available from the corresponding author upon reasonable request.
