# Supplementary material for: Effects of venlafaxine on the expression level and methylation status of genes involved in oxidative stress in rats exposed to a chronic mild stress
Source: J Cell Mol Med. 2020 Apr 13;24(10):5675–94. doi: 10.1111/jcmm.15231 (PMC7214168; doi:10.1111/jcmm.15231)
Supplement: Supplementary file 1 — Supplementary Material [file JCMM-24-5675-s001.docx]

1. **Supplementary information**

**Supplementary Table 1.** Characteristics of the genes studied (All data contained in the table were compiled with the help of Genomatix Software Suite, Intrexon Bioinformatics Germany GmbH, Munich, Germany, 2019).

| Gene name | Function of protein encoding the gene | Chromosomal location | alternative transcripts | promotors | SNPs affecting transcription factor binding sites | SNPs affecting protein sequence | 5’UTR | 3’UTR | repeat regions | exons |
| --- | --- | --- | --- | --- | --- | --- | --- | --- | --- | --- |
| Catalase (CAT) | hydrogen peroxide reductase | 3 | 1 | 3 | 6 | 2 | 1 | 1 | 8 | 13 |
| Glutathione peroxidase 1 (Gpx1) | Catalyses reduction of organic hydroperoxides and hydrogen peroxide by glutathione and thereby protect cells from oxidative damage | 8 | 1 | 1 | 0 | 1 | 1 | 1 | 0 | 2 |
| Glutathione peroxidase 4 (Gpx4) | Catalyses reduction of hydrogen peroxide, organic hydroperoxides and lipid hydroperoxides, and thereby protect cells from oxidative damage | 7 | 4 | 6 | 8 | 1 | 2 | 11 | 2 | 22 |
| superoxide dismutase 1 (SOD1) | Catalyzes conversion of supeoxide to hydrogen peroxide and molecular oxygen, involved in response to oxidative stress | 11 | 1 | 2 | 2 | 0 | 2 | 2 | 1 | 5 |
| Superoxide dismutase 2 (SOD2) | Intramitochondrial free radical scavenging enzyme | 1 | 1 | 2 | 2 | 0 | 1 | 1 | 1 | 5 |
| Nitric oxide synthase 1 (NOS1) | Catalyzes production of nitric oxide | 12 | 5 | 13 | 1 | 3 | 19 | 23 | 22 | 170 |
| Nitric oxide synthase 2 (NOS2) | Cytokine-inducible enzyme involved in nitric oxide production | 10 | 52 | 5 | 1 | 1 | 55 | 27 | 85 | 1383 |

**Supplementary Table 2.**

mRNA expression of Gpx1, Gpx4, CAT genes in PBMCs of animals exposed to CMS for two weeks (Control, Stressed) and in animals exposed to CMS for seven weeks and administered vehicle (1 ml/kg) or venlafaxine (10 mg/kg) for five weeks (Control/Venla, Stressed/Saline, Stressed/Venla). Relative gene expression levels were estimated using the 2^--ΔCt^ (Ct_gene_–Ct_18S_) method. The 2^-ΔCt^ values were re-calculated into relative copy number values (number of gene mRNA copies per 1 000 copies of 18S mRNA). Data represents means ± SEM. N = 6. No significant changes were found between any groups.

| Group | Gpx1 | Gpx4 | CAT |
| --- | --- | --- | --- |
| Control | 5.7*10^-4^ ± 2.0*10^-5^ | 4.9*10^-5^ ± 4.5*10^-7^ | 7.7*10^-5^ ± 1.7*10^-6^ |
| Stressed | 4.2*10^-4^ ± 1.3*10^-5^ | 4.9*10^-5^  ± 8.7*10^-7^ | 1.1*10^-4^ ± 1.7*10^-6^ |
| Control/Venla | 5.9*10^-4^ ± 1.9*10^-5^ | 4.3*10^-5^  ± 4.1*10^-7^ | 7.5*10^-5^ ± 1.9*10^-6^ |
| Stressed/Saline | 6.2*10^-4^ ± 2.6*10^-5^ | 5.3*10^-5^  ± 3.2*10^-7^ | 7.9*10^-5^ ± 1.9*10^-6^ |
| Stressed/Venla | 4.4*10^-4^ ± 5.3*10^-5^ | 8.8*10^-5^  ± 4.5*10^-7^ | 1.0*10^-4^ ± 1.1*10^-5^ |

**Supplementary Table 3.**

Methylation level of CAT promoter, Gpx4 promoter 2, Gpx4 promoter 3, SOD1 promoter, SOD2 promoter, NOS1 promoter 3 and NOS1 promoter 7 in PBMCs of animals exposed to CMS for two weeks (Control, Stressed) and in animals exposed to CMS for seven weeks and administered vehicle (1 ml/kg) or venlafaxine (10 mg/kg) for five weeks (Control/Venla, Stressed/Saline, Stressed/Venla). Data represents means ± SEM. N = 6. No significant changes were found between any groups.

| Group | CAT | Gpx4 | Gpx4 | SOD1 | SOD2 | NOS1 3 | NOS1 7 |
| --- | --- | --- | --- | --- | --- | --- | --- |
| Control | 51.50 ± 5.10 | 75.72 ± 24.28 | 51.50 ± 7.16 | 45.86 ± 4.50 | 32.65 ± 23.36 | 99.00 ± 0.58 | 61.52 ± 22.30 |
| Stressed | 55.65 ± 10.60 | 66.67 ± 33.33 | 55.65 ± 8.65 | 30.66 ± 13.37 | 13.88 ± 8.17 | 96.73 ± 3.27 | 66.34 ± 17.10 |
| Control/Venla | 53.62 ± 6.89 | 72.86 ± 23.47 | 52.11 ± 11.68 | 40.89 ± 9.82 | 29.18 ± 15.21 | 98.77 ± 2.11 | 62.15 ± 16.68 |
| Stressed/Saline | 61.02 ± 11.02 | 61.89 ± 27.89 | 65.72 ± 9.21 | 33.56 ± 11.65 | 19.21 ± 9.85 | 97.82 ± 3.82 | 65.48 ± 19.75 |
| Stressed/Venla | 70.06 ± 16.86 | 98.67 ± 0.88 | 70.15 ± 15.60 | 36.73 ± 12.45 | 26.31 ± 16.51 | 98.67 ± 0.89 | 61.21 ± 15.79 |

**Supplementary Table 4.**

Methylation level of CAT, Gpx1, Gpx4 promoter 2, Gpx4 promoter 3, SOD1, SOD2 promoter, NOS1 promoter 3 and NOS1 promoter 7 in hippocampus, amygdala, hypothalamus, midbrain, cortex and basal ganglia of animals exposed to CMS for two weeks (Control, Stressed) and in animals exposed to CMS for seven weeks and administered vehicle (1 ml/kg) or venlafaxine (10 mg/kg) for five weeks (Control/Venla, Stressed/Saline, Stressed/Venla). Data represents means ± SEM. N = 6. No significant changes were found between any groups.

| Methylation level of CAT promoter | | | | | |
| --- | --- | --- | --- | --- | --- |
| Structures | Control | Stressed | Control/Venla | Stressed/Saline | Stressed/Venla |
| Hippocampus | 65.13 ± 8.40 | 85.07 ± 2.21 | 55.13 ± 18.40 | 64.06 ± 7.10 | 52.89 ± 18.80 |
| Amygdala | 92.32 ± 0.85 | 93.30 ± 2.98 | 82.32 ± 10.23 | 63.49 ± 14.07 | 65.64 ± 6.82 |
| Hypothalamus | 61.73 ± 15.16 | 76.08 ± 16.08 | 65.51 ± 12.11 | 62.28 ± 0.21 | 66.48 ± 10.99 |
| Midbrain | 54.20 ± 23.04 | 51.78 ± 6.33 | 63.25 ± 18.15 | 70.47 ± 7.40 | 69.18 ± 21.99 |
| Cerebral cortex | 60.57 ± 8.02 | 74.94 ± 12.97 | 50.66 ± 12.22 | 37.57 ± 24.26 | 59.29 ± 14.01 |
| Basal ganglia | 91.38 ± 2.99 | 61.50 ± 5.31 | 81.22 ± 12.89 | 71.78 ± 7.87 | 74.52 ± 7.33 |

| Methylation level of Gpx1 promoter | | | | | |
| --- | --- | --- | --- | --- | --- |
| Structures | Control | Stressed | Control/Venla | Stressed/Saline | Stressed/Venla |
| Hippocampus | 88.65 ± 3.31 | 94.32 ± 9.42 | 75.34 ± 13.13 | 96.10 ± 0.75 | 59.38 ± 27.30 |
| Amygdala | 60.07 ± 22.01 | 57.68 ± 20.73 | 59.11 ± 6.71 | 73.85 ± 5.58 | 93.14 ± 17.21 |
| Hypothalamus | 12.27 ± 6.11 | 93.85 ± 11.97 | 15.42 ± 5.89 | 58.66 ± 8.59 | 52.61 ± 17.60 |
| Midbrain | 49.02 ± 11.98 | 86.96 ± 5.91 | 52.32 ± 9.63 | 93.95 ± 3.49 | 55.94 ± 23.98 |
| Cerebral cortex | 72.59 ± 14.26 | 55.33 ± 23.12 | 65.89 ± 17.38 | 56.09 ± 13.27 | 59.85 ± 12.75 |
| Basal ganglia | 67.15 ± 9.61 | 82.48 ± 10.11 | 71.42 ± 12.96 | 69.71 ± 13.92 | 93.21 ± 14.56 |

| Methylation level of Gpx4 promoter 2 | | | | | |
| --- | --- | --- | --- | --- | --- |
| Structures | Control | Stressed | Control/Venla | Stressed/Saline | Stressed/Venla |
| Hippocampus | 82.17 ± 14.56 | 49.12 ± 11.85 | 76.08 ± 19.53 | 51.97 ± 21.59 | 52.12+39.09 |
| Amygdala | 59.15 ± 33.36 | 52.42 ± 9.49 | 61.64 ± 19.71 | 54.43 ± 15.63 | 59.75 ± 32.86 |
| Hypothalamus | 85.60 ± 11.76 | 79.71 ± 16.87 | 87.07 ± 10.56 | 82.74 ± 19.83 | 89.37 ± 9.87 |
| Cerebral cortex | 99.00 ± 0.82 | 98.56 ± 0.94 | 99.60 ± 0.32 | 98.02 ± 0.89 | 98.75 ± 0.92 |

| Methylation level of Gpx4 promoter 3 | | | | | |
| --- | --- | --- | --- | --- | --- |
| Structures | Control | Stressed | Control/Venla | Stressed/Saline | Stressed/Venla |
| Hippocampus | 69.18 ± 12.77 | 90.54 ± 3.26 | 72.51 ± 9.87 | 86.20 ± 0.24 | 86.31 ± 6.00 |
| Amygdala | 76.37 ± 6.93 | 73.28 ± 5.77 | 79.63 ± 9.37 | 77.31 ± 2.22 | 75.57 ± 3.38 |
| Hypothalamus | 63.43 ± 14.70 | 85.21 ± 9.56 | 70.31 ± 11.02 | 77.50 ± 12.32 | 65.53 ± 11.21 |
| Midbrain | 86.29 ± 8.06 | 70.26 ± 9.16 | 79.11 ± 9.16 | 67.70 ± 7.11 | 51.70 ± 13.12 |
| Cerebral cortex | 74.07 ± 2.20 | 88.08 ± 24.11 | 71.51 ± 8.27 | 78.68 ± 1.22 | 86.82 ± 10.75 |
| Basal ganglia | 77.89 ± 11.81 | 84.23 ± 16.32 | 79.21 ± 9.81 | 75.58 ± 11.68 | 62.20 ± 15.21 |

| Methylation level of SOD1 promoter | | | | | |
| --- | --- | --- | --- | --- | --- |
| Structures | Control | Stressed | Control/Venla | Stressed/Saline | Stressed/Venla |
| Amygdala | 40.74 ± 7.05 | 55.47 ± 12.63 | 39.99 ± 11.32 | 56.38 ± 9.21 | 59.06 ± 13.43 |
| Hypothalamus | 25.95 ± 8.26 | 51.13 ± 1.34 | 35.11 ± 17.22 | 46.91 ± 3.82 | 50.26 ± 1.14 |
| Midbrain | 44.25 ± 5.01 | 18.65 ± 5.62 | 33.11 ± 11.89 | 23.39 ± 19.10 | 56.13 ± 3.93 |
| Cerebral cortex | 56.18 ± 7.85 | 52.29 ± 15.23 | 51.85 ± 18.21 | 47.12 ± 0.70 | 49.62 ± 4.07 |
| Basal ganglia | 43.72 ± 11.02 | 57.19 ± 5.81 | 51.21 ± 10.99 | 51.78 ± 1.46 | 58.48 ± 5.59 |

| Methylation level of SOD2 promoter | | | | | |
| --- | --- | --- | --- | --- | --- |
| Structures | Control | Stressed | Control/Venla | Stressed/Saline | Stressed/Venla |
| Amygdala | 37.31 ± 9.96 | 68.57 ± 21.84 | 41.15 ± 11.02 | 47.11 ± 24.55 | 65.69 ± 15.38 |
| Hypothalamus | 15.72 ± 12.84 | 43.02 ± 24.28 | 18.64 ± 9.85 | 39.56 ± 9.54 | 43.96 ± 23.19 |
| Midbrain | 27.96 ± 22.83 | 66.28 ± 27.53 | 31.69 ± 19.83 | 69.96 ± 31.09 | 75.09 ± 16.24 |
| Cerebral cortex | 34.35 ± 5.39 | 29.57 ± 14.80 | 32.35 ± 7.99 | 30.81 ± 14.07 | 38.51 ± 25.65 |
| Basal ganglia | 67.47 ± 15.83 | 74.26 ± 29.18 | 71.89 ± 21.08 | 90.96 ± 7.38 | 91.56 ± 6.89 |

| Methylation level of NOS1 promoter 3 | | | | | |
| --- | --- | --- | --- | --- | --- |
| Structures | Control | Stressed | Control/Venla | Stressed/Saline | Stressed/Venla |
| Hippocampus | 70.09 ± 4.09 | 75.21 ± 0.91 | 69.09 ± 9.91 | 74.67 ± 5.76 | 72.64 ± 8.99 |
| Amygdala | 80.70 ± 25.75 | 55.40 ± 16.99 | 79.89 ± 21.32 | 44.10 ± 1.93 | 71.01 ± 22.64 |
| Hypothalamus | 51.07 ± 15.82 | 69.08 ± 3.31 | 49.09 ± 17.21 | 42.60 ± 12.09 | 77.63 ± 22.32 |
| Midbrain | 31.17 ± 0.85 | 64.16 ± 29.26 | 39.81 ± 10.89 | 41.68 ± 3.03 | 45.19 ± 17.30 |
| Cerebral cortex | 67.06 ± 26.90 | 59.80 ± 23.02 | 59.09 ± 25.32 | 37.23 ± 2.75 | 40.68 ± 9.14 |
| Basal ganglia | 39.37 ± 15.55 | 58.96 ± 21.82 | 41.71 ± 17.71 | 86.76 ± 30.10 | 73.97 ± 20.67 |

| Methylation level of NOS1 promoter 7 | | | | | |
| --- | --- | --- | --- | --- | --- |
| Structures | Control | Stressed | Control/Venla | Stressed/Saline | Stressed/Venla |
| Hippocampus | 45.03 ± 9.11 | 53.43 ± 12.31 | 49.11 ± 11.21 | 43.57 ± 10.11 | 55.15 ± 11.70 |
| Amygdala | 51.87 ± 10.52 | 83.21 ± 38.99 | 49.78 ± 9.23 | 58.96 ± 16.87 | 49.9 ± 18.27 |
| Hypothalamus | 38.07 ± 12.79 | 27.94 ± 9.58 | 41.71 ± 13.99 | 25.16 ± 9.21 | 51.15 ± 19.76 |
| Midbrain | 57.51 ± 9.21 | 59.78 ± 15.63 | 51.41 ± 8.72 | 43.79 ± 17.01 | 42.48 ± 15.16 |
| Cerebral cortex | 68.04 ± 26.72 | 45.75 ± 12.29 | 59.01 ± 16.85 | 41.69 ± 11.99 | 37.28 ± 16.72 |
| Basal ganglia | 41.93 ± 13.97 | 55.71 ± 10.99 | 39.32 ± 15.22 | 54.01 ± 12.17 | 35.23 ± 19.36 |

**Supplementary Table 5.**

Differences in the methylation level of SOD1, SOD2 (A), NOS1 promoter 7 (B) between brain tissue and PBMCs of animals exposed to CMS for two weeks (Control, Stressed) and in animals exposed to CMS for seven weeks and administered vehicle (1 ml/kg) or venlafaxine (10 mg/kg) for five weeks (Control/Venla, Stressed/Saline, Stressed/Venla). Data represents means ± SEM. N = 6. No significant changes were found between any groups.

| Group | SOD1 promoter | | SOD2 promoter | | NOS1 promoter 7 | |
| --- | --- | --- | --- | --- | --- | --- |
|  | Brain | Blood | Brain | Blood | Brain | Blood |
| Control | 42.76 ± 1.69 | 45.86 ± 4.50 | 27.27 ± 8.95 | 32.65 ± 23.36 | 44.58 ± 5.53 | 61.52 ± 22.30 |
| Stressed | 47.56 ± 1.92 | 30.66 ± 13.37 | 21.43 ± 9.76 | 13.88 ± 8.17 | 53.52 ± 11.96 | 66.34 ± 17.10 |
| Control/Venla | 42.65 ± 5.36 | 40.89 ± 9.82 | 35.25 ± 8.42 | 29.18 ± 15.21 | 55.86 ± 6.85 | 62.15 ± 16.68 |
| Stressed/Saline | 35.87 ±7.99 | 33.56 ± 11.65 | 30.18 ± 6.43 | 19.21 ± 9.85 | 61.95 ± 7.65 | 65.48 ± 19.75 |
| Stressed/Venla | 53.18 ± 5.71 | 36.73 ± 12.45 | 35.52 ± 6.78 | 26.31 ± 16.51 | 53.53 ± 4.71 | 61.21 ± 15.79 |

**Supplementary Table 6.**

Expression of NOS2 and SOD1 proteins in animals exposed to CMS for two weeks (Control, Stressed) and in animals exposed to CMS for seven weeks and administered vehicle (1 ml/kg) or venlafaxine (10 mg/kg) for five weeks (Control/Venla, Stressed/Saline, Stressed/Venla). Samples containing 25 μg of proteins were resolved by SDS-PAGE. The intensity of the bands corresponding to NOS1 and SOD1 was analysed by densitometry, and integrated optical density (IOD) was normalized by protein content and a reference sample (see the Methods for details). The data show mean IODs of the bands from all analysed samples. The IOD_gene_/IOD_ACTB_ method was used to estimate the relative protein expression levels in the analysed samples.

N = 6. No significant changes were found between any groups.

| Expression of NOS2 proteins | | | | | |
| --- | --- | --- | --- | --- | --- |
| Structures | Control | Stressed | Control/Venla | Stressed/Saline | Stressed/Venla |
| Hippocampus | 3.008 ± 1.004 | 3.231 ± 1.347 | 1.908 ± 0.604 | 1.542 ± 0.905 | 2.335 ± 0.318 |
| Amygdala | 1.556 ± 0.673 | 2.933 ± 1.028 | 2.156 ± 0.503 | 2.607 ± 0.330 | 2.422 ± 0.668 |
| Hypothalamus | 3.680 ± 0.809 | 2.034 ± 0.695 | 2.990 ± 0.511 | 2.843 ± 0.663 | 2.123 ± 0.748 |
| Midbrain | 1.461 ± 0.682 | 1.917 ± 0.992 | 1.531 ± 0.786 | 2.015 ± 0.734 | 2.618 ± 0.634 |
| Cerebral cortex | 3.843 ± 1.613 | 1.509 ± 0.761 | 2.949 ± 1.010 | 1.798 ± 0.709 | 1.262 ± 0.301 |
| Basal ganglia | 2.165 ± 0.574 | 2.208 ± 0.120 | 1.991 ± 0.891 | 3.008 ± 0.723 | 2.001 ± 0.221 |

| Expression of SOD1 proteins | | | | | |
| --- | --- | --- | --- | --- | --- |
| Structures | Control | Stressed | Control/Venla | Stressed/Saline | Stressed/Venla |
| Hippocampus | 1.595 ± 0.604 | 2.811 ± 1.535 | 2.192 ± 0.711 | 2.430 ± 1.403 | 2.851 ± 0.958 |
| Amygdala | 1.618 ± 0.831 | 1.381 ± 0.719 | 1.337 ± 0.639 | 1.569 ± 0.168 | 2.254 ± 0.733 |
| Hypothalamus | 2.523 ± 1.281 | 1.325 ± 0.689 | 2.113 ± 0.963 | 1.496 ± 0.448 | 2.320 ± 0.769 |
| Midbrain | 3.566 ± 1.892 | 1.779 ± 0.928 | 2.986 ± 0.889 | 1.218 ± 0.619 | 1.279 ± 0.563 |
| Cerebral cortex | 4.178 ± 2.287 | 2.642 ± 1.461 | 3.178 ± 1.028 | 1.192 ± 0.990 | 1.985 ± 0.586 |
| Basal ganglia | 3.485 ± 1.895 | 1.830 ± 0.935 | 3.221 ± 1.002 | 1.375 ± 0.722 | 1.974 ± 0.672 |
